# Risk factors for the recurrence of chronic subdural hematomas in patients undergoing burr-hole drainage

**DOI:** 10.1007/s10143-026-04559-5

**Published:** 2026-11-09

**Authors:** Tabea Stoessel, Abeelan Rasadurai, Raphael Guzman, Jehuda Soleman

**Affiliations:** 1 Department of Neurosurgery, University Hospital Basel, Basel, Switzerland; 2 Faculty of Medicine, University of Basel, Basel, Switzerland

**Keywords:** Burr-hole drainage, Chronic subdural hematoma, cSDH, Midline shift, Recurrence, Risk factors

## Abstract

Chronic subdural hematoma (cSDH) is a common neurosurgical disease, particularly in the elderly. The recurrence of cSDH remains a clinical challenge, with several risk factors postulated. Although adjunctive treatments to surgery exist, such as steroids, statins, and middle meningeal artery embolization, the number needed to treat is high, and patient selection remains unclear. This retrospective study aimed to identify risk factors for recurrence after burr hole drainage of cSDH. A single-center retrospective study included consecutive patients undergoing burr-hole drainage for cSDH between February 2012 and May 2023. The primary outcome was surgical recurrence, defined as cSDH recurrence requiring reoperation within six months. Clinical characteristics, preoperative radiological findings, comorbidities and medications, and postoperative findings were assessed as potential risk factors using univariate analysis and, after imputation, multivariable analysis.A total of 766 patients with a recurrence rate of 11.2% (86/766) were included. Significant preoperative risk factors for recurrence included subacute and separated hematoma types (*p* = 0.02), larger hematoma volume (*p* = 0.004), greater midline shift (MLS) (*p* = 0.006), lower Glasgow Coma Scale (GCS) (*p* < 0.001) and higher Markwalder score (*p* = 0.012). Postoperative findings significantly associated with recurrence were larger cavity volume (*p* = 0.005), greater MLS (*p* = 0.01), a higher Markwalder score (*p* = 0.01) and modified Rankin Scale (mRS) score (*p* < 0.001) at discharge. After multivariable logistic regression, subacute hematoma age (OR 2.097, *p* = 0.036), absence of hypertension (OR 1.937, *p* = 0.012), larger postoperative cavity volume (OR 1.012, *p* = 0.016), greater postoperative MLS (OR 1.179, *p* < 0.001), and higher mRS at discharge (OR 1.959, *p* = 0.002) remained independently associated with recurrence. We found subacute hematoma, larger postoperative subdural cavity volume, higher mRS at discharge, greater postoperative MLS, and the absence of hypertension as factors significantly associated with surgical recurrence within six months.

**Supplementary Information:**

The online version contains supplementary material available at https://doi.org/10.1007/s10143-026-04559-5.

## Introduction

Chronic subdural hematoma (cSDH) is a common condition in neurosurgery, primarily affecting elderly patients following head trauma. The overall incidence in the Swiss population is approximately 8.2 per 100,000 per year. This incidence increases with age, reaching 64.2 per 100,000 per year in the 80–89 age group in Switzerland [1]. Due to medical advancements and increasing life expectancy, the incidence of cSDH is expected to rise in the coming years [2].

Surgical treatment remains the gold standard for cSDH, typically performed via burr hole drainage combined with subperiosteal drain insertion. Despite generally good outcomes, recurrence rates remain a concern and are reported between 7 and 20% across studies, with large randomized controlled trials (RCTs) indicating rates closer to 10% [3–8].

Further research has focused on strategies to reduce recurrence, including optimization of surgical techniques [3, 5, 6, 8–10] and the addition of adjuvant therapies such as pharmacological treatments (e.g., statins, corticosteroids) [11–17] or middle meningeal artery embolization (MMAE) [18–22]. While some of these approaches have shown promising results, their widespread use is limited by either high numbers needed to treat (e.g., for MMAE) or by potential adverse events (e.g., for corticosteroids).

Consequently, improved identification of patients at high risk for recurrence is crucial to enable more targeted application of these adjunctive therapies. Although various retrospective studies have investigated potential preoperative and postoperative risk factors for recurrence, the findings to date remain inconclusive [23–27].

The aim of this study is to analyze recurrence rates and associated preoperative and postoperative risk factors using univariate and multivariable methods. Ultimately, this study seeks to contribute to the development of a tool that could help predict recurrence risk in cSDH patients allowing more personalized treatment strategies with selective use of adjunct therapies and thereby reducing unnecessary exposure to radiation, costs, and the risk of adverse events.

## Methods

### Study design

In this study, we retrospectively analyzed the data of patients who underwent burr hole drainage for the evacuation of a cSDH at the Neurosurgical Department University Hospital Basel between February 2012 and May 2023. We included all patients above 18 years and excluded patients with the need for expansion of the surgery to a (mini-) craniotomy or with a documented refusal. The data were collected from the patient’s electronic medical and radiological files. No patients were excluded due to missing data.

Eligible patients were divided into two groups based on whether they developed a recurrent cSDH requiring reoperation during the first 6 months of follow-up (= “recurrent” group) or not (= “non-recurrent” group). Recurrence was defined as a reformation of cSDH collection leading to symptoms indicating surgical evacuation of the recurrent hematoma. Asymptomatic recurrence not indicating surgical treatment was not defined as recurrence, while acute subdural collection was defined as bleeding complication and not recurrence. By focusing on recurrence requiring reoperation, we aimed to identify factors with the highest clinical relevance. However, this definition deliberately excludes cases of radiographic recurrence that remained asymptomatic and were managed conservatively [28].

The primary outcome was surgical recurrence, defined as recurrence requiring reoperation within six months. To identify potential factors influencing recurrence, we examined four distinct categories: clinical characteristics, preoperative radiological findings, comorbidities and medication, postoperative findings. This categorical approach allowed for a broad evaluation of parameters associated with outcome, even in the absence of formally defined outcome measures. For the evaluation of the clinical presentation of the patients, we grouped the Markwalder Classification into “favorable” (Grade 0–1), “moderate” (Grade 2–3), and “severe” (Grade 4), the modified Rankin Scale (mRS) into “favorable” (mRS 0–2) and “non-favorable” (mRS 3–5) as well as Glasgow Coma Scale (GCS) into “favorable” (GCS 14–15), “moderate” (GCS 9–13) and “severe” (GCS < 9).

Since the surgical procedure itself followed a highly standardized protocol throughout the study period, intraoperative factors were not included in the analysis. The only exception was the type of drain placement, as our institutional practice evolved from subdural to predominantly subperiosteal drainage; therefore, this parameter was recorded, but no broader intraoperative comparison was performed.

This study was conducted in accordance with the ethical principles outlined in the Declaration of Helsinki and its subsequent amendments. Ethical approval for this retrospective study was obtained from the local Ethics Committee of Northwestern and Central Switzerland (EKNZ; Project-ID 2025 − 00588). Given the retrospective design and the use of fully anonymized clinical and radiological data, the requirement for written informed consent was waived by the ethics committee. All patient data were handled in compliance with applicable data protection regulations.

### Surgical management

The surgical indication was determined according to the internal standard of the University Hospital Basel [29]. An absolute indication for surgery exists in patients with a cSDH showing a width of more than one calvarial thickness (> 10 mm) and/or a midline shift (MLS) of > 5 mm accompanied by neurological deficits. A relative indication for surgery exists in patients with a cSDH of similar dimensions (> 10 mm and/or > 5 mm MLS) but without neurological deficits.

All patients who underwent surgery were preoperatively diagnosed with a symptomatic cSDH. The surgical procedure consisted of a unilateral or, if necessary, bilateral burr hole trepanation using one or two burr holes (6.1% vs. 93.9%, respectively), performed under general anesthesia with the patient in supine position. After opening the dura, the hematoma was irrigated using warm normal saline. At the end of the procedure, a ventilation maneuver was performed to prevent postoperative gas accumulation and residual hematoma formation. A drain was placed in all patients, either in subdural or subperiosteal fashion (non-recurrent group 37.9% vs. 62.1% and recurrent group 46.3% vs. 53.7%, respectively) and was removed within 48 h postoperatively.

### Preoperative management

Preoperative management followed a standardized institutional protocol of the University Hospital Basel. All patients underwent a structured clinical and radiological assessment before surgery. The following preoperative variables were collected and evaluated: neurological status (e.g., GCS, Markwalder score, presence of motor, sensory, or speech deficits, cognitive decline, seizures), hematoma characteristics on imaging (hematoma age and texture according to the Nakaguchi classification, presence of layering, volumetric hematoma assessment, and MLS), as well as comorbidities and medication status (e.g., Charlson Comorbidity Index, hypertension, diabetes, renal or hepatic disease, antithrombotic therapy including antiplatelets, vitamin-K antagonists, direct oral anticoagulants (DOACs), and perioperative interruption times) [24].

### Postoperative management

In a total of 622 patients a computed tomography (CT) scan was performed postoperatively either within 48 h (*n* = 88) or during the follow-up period (*n* = 323) or during both periods (*n* = 211), depending on the patient’s neurological symptoms and the surgeon’s discretion. This imaging was used to assess cavity volume, the presence of pneumocephalus, the extent of MLS, and any recurrent hematomas, such as acute subdural hematomas (aSDH) and cSDH. Based on the clinic and the CT findings, a recommendation for reoperation of the cSDH was made.

### Statistical analysis

A descriptive data analysis was performed to summarize the characteristics of the dataset. Univariate analyses were conducted using the Chi-square test for categorical variables presented as number of patients and percentage and the Wilcoxon test for continuous variables presented as mean with standard deviation and median with interquartile range (IQR). To evaluate a broad range of potential risk factors while focusing on clinically and radiologically established parameters, all variables showing significance in the univariate analysis were further analyzed through a multivariable logistic regression analysis. For this step, missing values were imputed using missForest [30]. To mitigate small-sample bias and improve parameter estimation in the presence of a limited number of events, we employed Firth penalized logistic regression. While this method reduces estimation bias, it does not eliminate the risk of overfitting, correct for data-driven variable selection, or substitute for formal internal or external validation. Model performance was assessed using the C-statistic for apparent discrimination and the Hosmer-Lemeshow test to evaluate potential lack of fit [31, 32]. A forest plot analysis was performed to visualize the multivariable results. The final outputs included odds ratios (OR), standard errors, 95% confidence intervals (CI), and p-values to assess the independent association of each variable with cSDH recurrence. The significance level was set at *p* < 0.05. Finally, to evaluate the robustness of the imputation, a sensitivity analysis using a complete-case approach was performed.

Data management was carried out using REDCap (Research Electronic Data Capture) within a GDPR-compliant framework [33]. All analyses were performed using RStudio for Windows (Team (2020). RStudio: Integrated Development for R. RStudio, PBC, Boston, MA URL).

## Results

### Baseline

A total of 766 patients, with a mean age of 76.3 ± 11.1 years (27–97 years), were included in this study. The baseline characteristics of both groups are described in Table 1, showing balanced distribution amongst the two groups.

**Table 1:** Descriptive statistics of baseline characteristics

	Non-Recurrent (*N* = 680)	Recurrent (*N* = 86)	*P*-value
Sex			
Male	473 (69.6%)	68 (79.1%)	0.089
Female	207 (30.4%)	18 (20.9%)	
Age Group			
0–50	20 (2.9%)	2 (2.3%)	0.921
51–60	46 (6.8%)	6 (7.0%)	
61–70	83 (12.2%)	7 (8.1%)	
71–80	225 (33.1%)	31 (36.0%)	
81–90	262 (38.5%)	34 (39.5%)	
91–100	44 (6.5%)	6 (7.0%)	
Blood Thinner Use			
Yes	342 (50.3%)	46 (53.5%)	0.735
No	322 (47.4%)	39 (45.3%)	
Missing	16 (2.4%)	1 (1.2%)	
Preoperative Hemoglobin (g/L)			
0–80; Severe Anemia	6 (0.9%)	0 (0%)	0.473
80–110; Moderate Anemia	84 (12.4%)	8 (9.3%)	
110–125; Mild Anemia	128 (18.8%)	21 (24.4%)	
>125; Normal	449 (66.0%)	57 (66.3%)	
Missing	13 (1.9%)	0 (0%)	
Affected Hemisphere			
Left	241 (35.4%)	22 (25.6%)	0.185
Right	232 (34.1%)	30 (34.9%)	
Bilateral (Left> Right)	114 (16.8%)	21 (24.4%)	
Bilateral (Right> Left)	93 (13.7%)	13 (15.1%)	

### Clinical characteristics

The preoperative clinical characteristics of both groups are presented in Table 2. A significant difference was observed in the preoperative GCS, with the non-recurrent group showing more frequently a favorable GCS (85.6% vs. 73.3%, *p* < 0.001). Additionally, a significant difference was found in the preoperative Markwalder score (*p* = 0.012), with patients in the recurrent group showing higher rates of moderate Markwalder score grade (57.0% vs. 40.4%).

**Table 2:** Descriptive statistics of clinical characteristics

	Non-Recurrent (*N* = 680)	Recurrent (*N* = 86)	*P*-value
Preoperative GCS Group			
13–15	582 (85.6%)	63 (73.3%)	**< 0.001**
9–12	71 (10.4%)	21 (24.4%)	
<8	26 (3.8%)	2 (2.3%)	
Missing	1 (0.1%)	0 (0%)	
Preoperative Markwalder Group			
Grade 0–1	400 (58.8%)	37 (43.0%)	**0.012**
Grade 2–3	275 (40.4%)	49 (57.0%)	
Grade 4	4 (0.6%)	0 (0%)	
Missing	1 (0.1%)	0 (0%)	
Motor Deficit			
Yes	456 (67.1%)	54 (62.8%)	0.491
No	223 (32.8%)	32 (37.2%)	
Missing	1 (0.1%)	0 (0%)	
Sensory Deficit			
Yes	83 (12.2%)	10 (11.6%)	1
No	595 (87.5%)	76 (88.4%)	
Missing	2 (0.3%)	0 (0%)	
Speech Deficit			
Yes	221 (32.5%)	27 (31.4%)	0.926
No	458 (67.4%)	59 (68.6%)	
Missing	1 (0.1%)	0 (0%)	
Headache			
Yes	263 (38.7%)	39 (45.3%)	0.287
No	416 (61.2%)	47 (54.7%)	
Missing	1 (0.1%)	0 (0%)	
Cognitive Decline			
Yes	245 (36.0%)	36 (41.9%)	0.353
No	434 (63.8%)	50 (58.1%)	
Missing	1 (0.1%)	0 (0%)	
Seizure			
Yes	48 (7.1%)	6 (7.0%)	1
No	629 (92.5%)	80 (93.0%)	
Missing	3 (0.4%)	0 (0%)	

### Preoperative radiological findings

Analysis of preoperative radiological findings revealed a significant difference in hematoma age (*p* = 0.038) and hematoma characteristics (*p* = 0.02), with significantly higher rates of subacute (25.6% vs. 14.0%) and separated hematomas (27.9% vs. 16.8%) in the recurrent group. Additionally, the mean preoperative hematoma volume (158 ml vs. 128 ml, *p* = 0.004) and the preoperative MLS (22.50 mm vs. 7.12 mm, *p* = 0.002) were significantly larger in the recurrent group (Table 3).

**Table 3:** Descriptive statistics of preoperative radiological findings

	Non-Recurrent (*N* = 680)	Recurrent (*N* = 86)	*P*-value
Hematoma Age			
Chronic	258 (37.9%)	26 (30.2%)	**0.038**
Acute on Chronic	306 (45.0%)	35 (40.7%)	
Subacute	95 (14.0%)	22 (25.6%)	
Hygroma	18 (2.6%)	3 (3.5%)	
Missing	3 (0.4%)	0 (0%)	
Hematoma Characteristics			
Homogenous	224 (32.9%)	33 (38.4%)	**0.02**
Laminar	87 (12.8%)	9 (10.5%)	
Separated	114 (16.8%)	24 (27.9%)	
Trabecular	218 (32.1%)	17 (19.8%)	
Missing	37 (5.4%)	3 (3.5%)	
Layering			
Yes	193 (28.4%)	26 (30.2%)	0.878
No	462 (67.9%)	58 (67.4%)	
Missing	25 (3.7%)	2 (2.3%)	
Preoperative Hematoma Volume (ml)			
Mean (SD)	128 (79.5)	158 (140)	**0.004**
Median [Min, Max]	115 [0, 1120]	144 [48.0, 1260]	
IQR	76.25–160.75	92–181	
Missing	106 (15.6%)	9 (10.5%)	
Preoperative Midline Shift (mm)			
Mean (SD)	7.24 (9.47)	8.46 (5.14)	**0.006**
Median [Min, Max]	6.00 [0, 204]	8.00 [0, 25.0]	
IQR	4–10	5–11	
Missing	36 (5.3%)	5 (5.8%)	

### Comorbidity characteristics and medication intake

The analysis of the Charlson Comorbidity Index (CCI) revealed a significant difference between the groups, with patients in the recurrent group having a higher average CCI score (6.28 ± 2.53 vs. 5.72 ± 2.56, *p* = 0.022). A significant difference was also observed in the prevalence of hypertension, which was more common in the non-recurrent group (58.4% vs. 44.2%, *p* = 0.017) (Table 4).

**Table 4:** Descriptive statistics of comorbidity characteristics and medication intake

	Non-Recurrent (*N* = 680)	Recurrent (*N* = 86)	*P*-value
Charlson Comorbidity Index Score			
Mean (SD)	5.72 (2.56)	6.28 (2.53)	**0.022**
Median [Min, Max]	5.00 [0, 16.0]	6.00 [1.00, 16.0]	
IQR	4–7	5–8	
Charlson Comorbidity Index Group			
0	1 (0.1%)	0 (0%)	0.267
1–2	55 (8.1%)	5 (5.8%)	
3–4	174 (25.6%)	15 (17.4%)	
≥5	450 (66.2%)	66 (76.7%)	
Hypertension (HTN)			
Yes	397 (58.4%)	38 (44.2%)	**0.017**
No	283 (41.6%)	48 (55.8%)	
Diabetes Mellitus (DM)			
Yes	120 (17.6%)	18 (20.9%)	0.55
No	560 (82.4%)	68 (79.1%)	
Liver Cirrhosis			
Yes	15 (2.2%)	2 (2.3%)	1
No	663 (97.5%)	84 (97.7%)	
Missing	2 (0.3%)	0 (0%)	
Renal Insufficiency			
Yes	115 (16.9%)	17 (19.8%)	0.619
No	563 (82.8%)	69 (80.2%)	
Missing	2 (0.3%)	0 (0%)	
Coronary Heart Disease (KHK)			
Yes	169 (24.9%)	29 (33.7%)	0.101
No	511 (75.1%)	57 (66.3%)	
Peripheral Artery Disease (PAVK)			
Yes	24 (3.5%)	1 (1.2%)	0.4
No	656 (96.5%)	85 (98.8%)	
Atrial Fibrillation (VHF)			
Yes	169 (24.9%)	15 (17.4%)	0.167
No	511 (75.1%)	71 (82.6%)	
Hypercholesterolemia			
Yes	155 (22.8%)	26 (30.2%)	0.163
No	525 (77.2%)	60 (69.8%)	
BMI Group (kg/m^2)			
<18.5; Underweight	24 (3.5%)	5 (5.8%)	0.501
18.5–24.9; Normal weight	293 (43.1%)	40 (46.5%)	
>24.9; Overweight	348 (51.2%)	41 (47.7%)	
Missing	15 (2.2%)	0 (0%)	
Smoker			
Yes	100 (14.7%)	12 (14.0%)	0.981
No	580 (85.3%)	74 (86.0%)	
Blood Thinner Use			
Yes	342 (50.3%)	46 (53.5%)	0.735
No	322 (47.4%)	39 (45.3%)	
Missing	16 (2.4%)	1 (1.2%)	
Aspirin Use			
Yes	161 (23.7%)	28 (32.6%)	0.11
No	181 (26.6%)	18 (20.9%)	
Missing	338 (49.7%)	40 (46.5%)	
Marcoumar Use			
Yes	124 (18.2%)	15 (17.4%)	0.738
No	217 (31.9%)	31 (36.0%)	
Missing	339 (49.9%)	40 (46.5%)	
Plavix Use			
Yes	34 (5.0%)	6 (7.0%)	0.701
No	307 (45.1%)	40 (46.5%)	
Missing	339 (49.9%)	40 (46.5%)	
DOAC Use			
Yes	57 (8.4%)	5 (5.8%)	0.419
No	283 (41.6%)	41 (47.7%)	
Missing	340 (50.0%)	40 (46.5%)	
Number of Days with a Blood Thinner Stop Preoperative			
Mean (SD)	8.71 (21.7)	6.43 (15.6)	0.194
Median [Min, Max]	2.00 [0, 249]	1.00 [0, 77.0]	
IQR	1–6.5	0.38–2.00	
Missing	338 (49.7%)	40 (46.5%)	
Number of Days with a Blood Thinner Stop Postoperative			
Mean (SD)	35.6 (37.8)	33.0 (54.4)	0.138
Median [Min, Max]	28.0 [0, 275]	11.0 [0, 278]	
IQR	7–51	4.5–41.5	
Missing	414 (60.9%)	55 (64.0%)	
Total Number of Days with a Blood Thinner Stop			
Mean (SD)	43.2 (42.2)	41.2 (58.4)	0.147
Median [Min, Max]	33.5 [0, 286]	19.0 [0, 278]	
IQR	12–57	6.5–52	
Missing	414 (60.9%)	55 (64.0%)	
Conversion of Blood Thinner			
Yes	124	16	0.808
No	229	32	
Missing	327 (48.1%)	38 (44.2%)	
Steroid Use			
Yes	31 (4.6%)	6 (7.0%)	0.464
No	645 (94.9%)	79 (91.9%)	
Missing	4 (0.6%)	1 (1.2%)	
ACE Inhibitor Use			
Yes	136 (20.0%)	17 (19.8%)	1
No	543 (79.9%)	68 (79.1%)	
Missing	1 (0.1%)	1 (1.2%)	
Statin Use			
Yes	210 (30.9%)	30 (34.9%)	0.534
No	469 (69.0%)	56 (65.1%)	
Missing	1 (0.1%)	0 (0%)	

### Postoperative characteristics

Laboratory, morphological imaging, and clinical postoperative parameters are summarized in Table 5.

**Table 5:** Descriptive statistics of postoperative findings

	Non-Recurrent (*N* = 680)	Recurrent (*N* = 86)	*P*-value
Hb Postoperative			
Mean (SD)	120 (20.5)	120 (17.9)	0.724
Median [Min, Max]	121 [11.0, 172]	122 [76.0, 162]	
IQR	108–134	106–133	
Missing	207 (30.4%)	10 (11.6%)	
Tc Postoperative			
Mean (SD)	242 (104)	242 (136)	0.362
Median [Min, Max]	231 [16.0, 760]	213 [46.0, 807]	
IQR	169–301	152.25–277.5	
Missing	211 (31.0%)	12 (14.0%)	
INR Postoperative			
Mean (SD)	1.14 (0.179)	1.13 (0.119)	0.515
Median [Min, Max]	1.10 [0.700, 3.50]	1.10 [0.900, 1.70]	
IQR	1.1–1.2	1.1–1.2	
Missing	287 (42.2%)	20 (23.3%)	
Postoperative Cavity Volume (ml)			
Mean (SD)	52.9 (38.0)	74.1 (54.6)	**0.005**
Median [Min, Max]	44.0 [0, 238]	67.0 [4.00, 314]	
IQR	27–70	8–90.75	
Missing	420 (61.8%)	48 (55.8%)	
Postoperative Midline Shift in mm			
Mean (SD)	3.38 (3.81)	8.97 (20.8)	**0.01**
Median [Min, Max]	3.00 [0, 41.0]	5.00 [0, 129]	
IQR	1–5	1–7.75	
Missing	419 (61.6%)	48 (55.8%)	
Postoperative Pneumocephalus			
Yes	250 (36.8%)	37 (43.0%)	0.312
No	430 (63.2%)	49 (57.0%)	
Missing	424 (62.4%)	49 (57.0%)	
aSDH in Postoperative Imaging			
Yes	49	25	0.599
No	11	4	
Missing	220 (28.7%)	1 (1.2%)	
GCS Group at Release Date			
13–15	633 (93.1%)	74 (86.0%)	**0.042**
9–12	33 (4.9%)	7 (8.1%)	
<8	14 (2.1%)	5 (5.8%)	
Markwalder Group at Release Date			
0–1	583 (85.7%)	64 (74.4%)	**0.01**
2–3	84 (12.4%)	17 (19.8%)	
4	13 (1.9%)	5 (5.8%)	
mRS Score at Release Date			
Mean (SD)	1.51 (1.35)	2.31 (1.49)	**< 0.001**
Median [Min, Max]	1.00 [0, 6.00]	2.00 [0, 6.00]	
IQR	1–2	1–3	
mRS Group at Release Date			
0–2	515 (75.7%	50 (58.1%)	**< 0.001**
3–5	154 (22.6%)	31 (36.0%)	
6	11 (1.6%)	5 (5.8%)	

For the postoperative morphological imaging findings, only CT and MRI scans performed within 48 h after surgery were used. It was demonstrated that the postoperative cavity volume in the recurrent group was significantly larger than in the non-recurrent group (74.1 ml vs. 52.9 ml, *p* = 0.005). Similarly, postoperative MLS was greater in patients in the recurrent group (8.97 mm vs. 3.38 mm, *p* = 0.01).

The functional outcomes showed that patients in the recurrent group had poorer neurological scores at discharge. Looking at the GCS group, moderate and severe scores were more common in the recurrent group (moderate: 8.1% vs. 4.9%, severe: 5.8% vs. 2.1% *p* = 0.042). The Markwalder score was significantly worse in the recurrent group (*p* = 0.01), the mRS score at discharge significantly higher in the recurrent group (2.31 vs. 1.51, *p* < 0.001), while the recurrent group showed significantly more patients with non-favorable mRS at discharge (36.0% vs. 22.6%, *p* < 0.001).

### Multivariable logistic regression

**Fig. 1:**
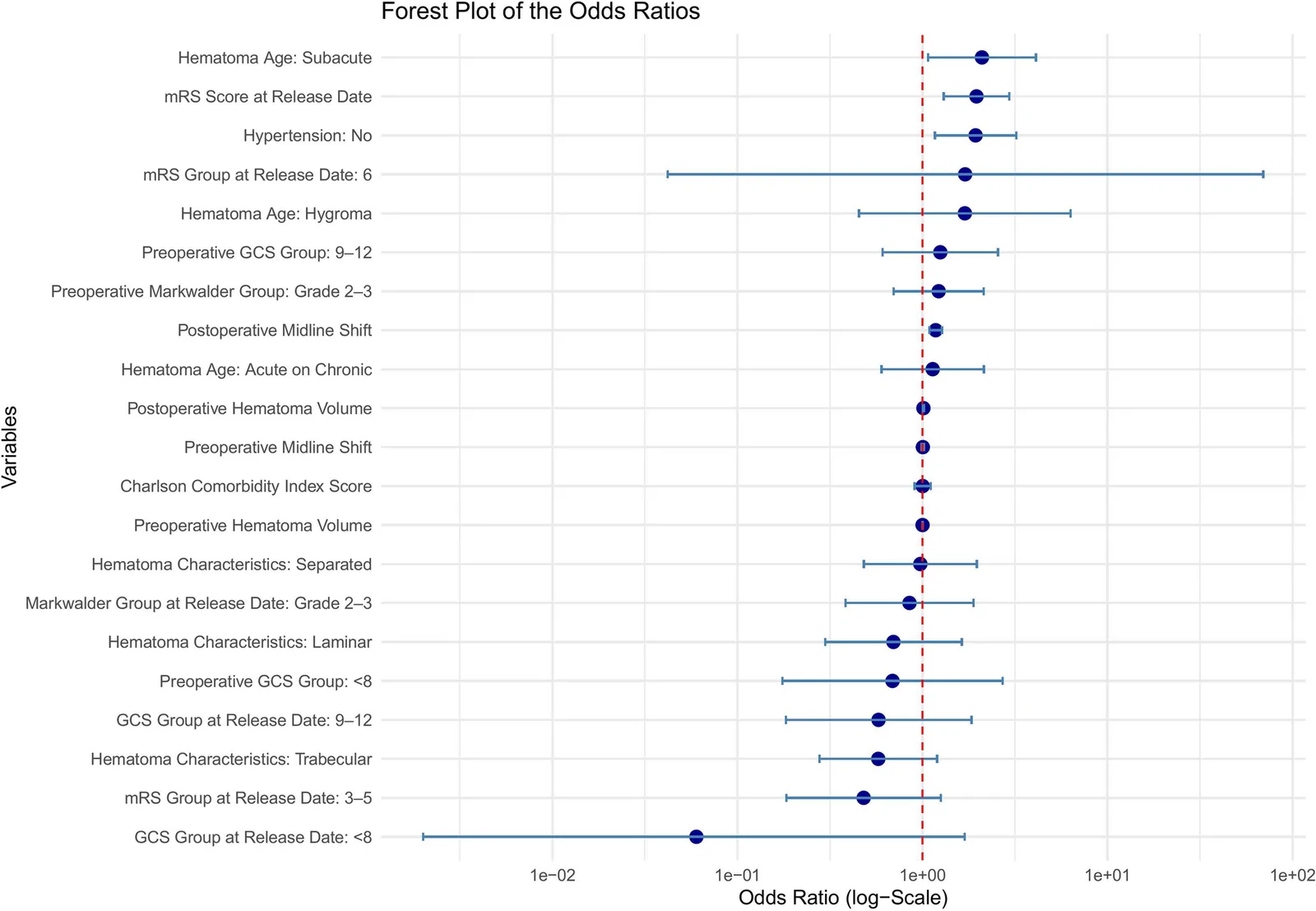
Forest plot illustrating the odds ratios of the multivariable logistic regression: Each horizontal line represents one variable included in the analysis. The blue dots indicate the estimated odds ratio (OR), while the horizontal lines denote the 95% confidence intervals (CI). Odds ratios are shown on a logarithmic scale. Values to the left of the red dashed line (OR < 1) suggest a protective association, while values to the right (OR > 1) indicate an increased risk. The vertical red dashed line at OR = 1 marks the point of no effect. Variables with confidence intervals crossing this line are not statistically significant.

After multivariate logistic regression analysis the absence of hypertension (OR = 1.937, *p* = 0.012), subacute hematoma age (OR = 2.097, *p* = 0.036), higher mRS at date of discharge (OR = 1.959, *p* = 0.002), both larger postoperative cavity volume (OR = 1.012, *p* = 0.016) and greater postoperative MLS (OR = 1.179, *p* < 0.001) remained, predictors of recurrence (Fig. 1; Table 6). The model demonstrated good apparent discrimination in the development dataset with an area under the receiver operating characteristic curve (AUC) of 0.809, and the Hosmer–Lemeshow test detected no significant evidence of lack of fit (*p* = 0.534). The complete-case sensitivity analysis (comprising 263 patients with 33 surgical recurrences) demonstrated an apparent discrimination AUC of 0.746 (95% CI: 0.645–0.847) and a Hosmer–Lemeshow test p-value of 0.400. While postoperative midline shift retained independent statistical significance (OR = 1.032 [95% CI 1.004–1.060], *p* = 0.018) and effect directions remained aligned, statistical significance was not retained for other primary predictors, including postoperative cavity volume and discharge mRS, due to the reduced sample size (Supplementary Table S1).

**Table 6:** Multivariable logistic regression of risk factor analysis for hematoma recurrence in cSDH patients

	Odds Ratios	std. Error	CI	*P*-value
(Intercept)	0.007	0.533	0.002–0.019	<0.001
Hypertension: No	1.937	0.258	1.167–3.213	**0.012**
Charlson Comorbidity Index Score	1.003	0.051	0.908–1.108	0.955
Preoperative GCS Group				
9–12	1.249	0.366	0.609–2.561	0.557
<8	0.689	0.7	0.175–2.715	0.601
Preoperative Markwalder Group: Grade 2–3	1.224	0.286	0.699–2.145	0.493
Preoperative Hematoma Volume	1.001	0.001	0.999–1.003	0.358
Preoperative Midline Shift	1.005	0.008	0.989–1.021	0.589
Hematoma Age				
Acute on Chronic	1.136	0.325	0.601–2.147	0.703
Subacute	2.097	0.342	1.072–4.102	**0.036**
Hygroma	1.695	0.672	0.454–6.324	0.458
Hematoma Characteristics				
Laminar	0.697	0.434	0.298–1.632	0.409
Separated	0.974	0.359	0.482–1.967	0.942
Trabecular	0.577	0.373	0.278–1.199	0.148
Postoperative Cavity Volume	1.012	0.005	1.003–1.021	**0.016**
Postoperative Midline Shift	1.179	0.04	1.09–1.274	**< 0.001**
GCS Group at Release Date				
9–12	0.579	0.589	0.183–1.84	0.363
<8	0.06	1.708	0.002–1.692	0.071
Markwalder Group at Release Date: Grade 2–3	0.851	0.406	0.384–1.888	0.670
mRS Group at Release Date				
3–5	0.481	0.491	0.184–1.258	0.148
6	1.701	1.893	0.042–69.495	0.782
Higher mRS Score at Release Date	1.959	0.208	1.304–2.944	**0.002**

## Discussion

In this study, we analyzed 766 consecutive patients who underwent burr-hole drainage for chronic subdural hematoma, comparing those who experienced recurrence within six months with those who did not, in order to identify potential risk factors associated with recurrence. A broad range of clinical, radiological, and comorbidity-related risk factors were evaluated [23–27, 38].

The recurrence rate of 11.2% in our cohort compares favorably with historical series (14–20% in the early 2000s) [3, 5, 7], while declining to 7.1% in recent years (2020–2024), reflecting the impact of standardized surgical techniques and treatment of patients within the framework of prospective standardized trials [6, 8]. These improvements likely attenuate the influence of previously reported risk factors. Accordingly, anticoagulant use which is historically associated with recurrence, showed no effect in our cohort (*p* = 0.735) or in a recent RCT, highlighting the need for contemporary, technique-adjusted risk stratification [7, 8]. This approach may improve the targeted use of adjuvant therapies such as MMAE or medical treatment in patients with persistently elevated residual risk [18–20].

It is important to note that our primary endpoint was surgical recurrence. While this provides a clear and clinically meaningful outcome, avoiding the inclusion of minor, asymptomatic collections, it may underestimate the true biological recurrence rate. Factors identified in this study are therefore specific predictors of reoperation rather than factors for purely radiographic recurrence, which might be managed conservatively.

Compared with the existing literature, our study is distinguished by its large single-center cohort and the comprehensive evaluation of baseline, preoperative, and postoperative parameters. Although several predictive models and nomograms have previously been developed to estimate recurrence risk, they are often based on smaller or historical cohorts. Many prior studies focused on isolated domains, such as radiological features or antithrombotic therapy, whereas our integrated multivariable approach may explain discrepancies between historically reported and independently significant predictors in our cohort [7, 24–27, 34–37]. A further strength is the highly standardized surgical management applied throughout the study period, minimizing procedural variability previously implicated in recurrence [5, 7]. This consistency allowed postoperative radiological markers, particularly residual cavity volume (OR = 1.012 per ml increase, *p* = 0.016) and postoperative MLS (OR = 1.179 per mm increase, *p* < 0.001), to emerge as the dominant independent predictors. While our findings do not directly evaluate whether adjunctive therapies improve outcomes in these patients, they may help identify high-risk candidates for evaluation in future prospective studies of postoperative adjunctive treatment (such as middle meningeal artery embolization [MMAE] or pharmacological therapies). In addition, part of the dataset was collected within the framework of RCTs conducted at our institution (cSDH-Drain Trial, SECA trial), enhancing data quality compared with purely retrospective series [6, 8]. Our results confirm the findings of the randomized trial by Santarius et al. and the more recent multicenter FINISH and SECA trial, which similarly showed that standardized surgical techniques and specific medication protocols lead to lower and more predictable recurrence rates than previously reported in older retrospective series [3, 5, 8].

Consistent with prior literature, subacute hematoma age was independently associated with recurrence (OR = 2.097, *p* = 0.036) [23–27]. Although separated hematomas were significant in univariate analysis (*p* = 0.02), this association did not persist after adjustment, despite their frequent identification as risk factors in previous studies [23, 26, 27, 38]. Volumetric assessment proved more informative than linear measurements, with postoperative cavity volume emerging as a significant predictor, suggesting that larger residual volumes may warrant closer surveillance or adjunctive treatment.

MLS remained a robust predictor both preoperatively (*p* = 0.006) and postoperatively (*p* = 0.01). In contrast, clinical severity scores (GCS, Markwalder) did not emerge as independent predictors in our multivariable analysis, suggesting that their impact is primarily reflected through the underlying radiological disease burden [27, 39].

Interestingly, the absence of hypertension was independently associated with recurrence in our multivariable analyses (OR = 1.937, *p* = 0.012). This finding contrasts with earlier reports linking hypertension to increased recurrence risk [7, 26]. While the underlying mechanism remains unclear, this association was not explained by antihypertensive medication use and most likely represents residual confounding, potential selection bias or a chance association within our study population. Although we examined the use of antihypertensive medications (e.g., ACE inhibitors), our study might have been underpowered to detect subtle medication-specific effects that could influence this association. Even though a higher comorbidity burden was observed in recurrent cases (*p* = 0.022), this did not persist after multivariable adjustment.

Overall, our findings align with a growing consensus that, in the context of standardized contemporary surgery, recurrence risk in cSDH is primarily driven by radiological indicators of residual disease rather than isolated clinical or medication-related factors. This may explain why results from older, heterogeneous cohorts are not fully transferable to modern practice.

### Future research

The present study serves as a foundational step in identifying key independent predictors of recurrence in a contemporary cohort. However, several limitations regarding our model’s performance must be acknowledged. First, the reported AUC of 0.809 represents apparent discrimination calculated within the development dataset without bootstrap correction, cross-validation, or independent external validation. The absence of a calibration plot and formal internal validation requires that our predictive findings remain preliminary. To further substantiate our findings and make more precise statements, larger studies are needed. Our group plans to utilize machine learning methods to assess the identified risk factors in greater detail. Further internal validation based on a single center prospective cohort and external validation based on a retrospective multicenter cohort are planned. Based on these results, a potential recurrence probability score will be developed, which will then be further validated in multicenter prospective studies. In parallel, future investigations should focus on the standardization of surgical techniques and a systematic evaluation of individual procedural steps, as variability in operative practice may substantially influence recurrence rates. With this, we aim to support improved risk stratification and individualized patient-specific therapy planning, with the targeted use of adjuvant therapies such as MMAE or medical agents in the future.

### Limitations

The primary limitations of this study are its retrospective design, and the inherent biases associated with data collection from medical records. While we employed advanced imputation techniques (missForest) to address missing data, the substantial missingness in postoperative radiological and medication-related variables (up to 60%) remains a limitation. This missingness primarily reflects clinical practice, where postoperative imaging was not mandated for asymptomatic patients. Consequently, the complete-case sensitivity analysis is inherently limited as postoperative imaging was obtained selectively according to clinical symptoms, surgeon judgment, or trial participation rather than through a uniform, mandatory imaging protocol. This non-random missingness must be carefully considered when interpreting the complete-case results. While the complete-case analysis showed aligned effect directions, several predictors lost statistical significance due to the reduced sample size, underscoring the reliance on imputed data. Additionally, postoperative imaging protocols were not standardized, as follow-up scans were performed based on clinical judgment or other ongoing randomized controlled studies rather than a predefined schedule, potentially introducing variability in the assessment of radiological outcomes [6, 8]. Another limitation is the single-center nature of this study, which may limit the generalizability of our findings due to institution-specific surgical practices and patient demographics.

Furthermore, our definition of recurrence was limited to cases requiring surgical intervention. Consequently, our results reflect predictors of clinical failure requiring reoperation and cannot be generalized to radiographic recurrences that do not necessitate further surgery. This distinction is crucial, as some risk factors might influence hematoma regrowth without necessarily leading to symptomatic recurrence or surgical indication.

## Conclusion

Based on our retrospective cohort of patients with cSDH undergoing burr-hole drainage, larger hematoma/cavity volume and greater MLS postoperatively, subacute hematomas, the absence of hypertension, as well as higher mRS Score at discharge are significantly associated with surgical recurrence within six months. Serving as a foundational step, **f**urther studies validating our results through machine learning or larger multicenter cohorts are needed to enable individualized patient-specific therapy for patients with cSDH.

## Supplementary Information

Below is the link to the electronic supplementary material.

Supplementary Material 1

## Data Availability

All data supporting the findings of this study are available within the paper. The raw study data is stored at the University Hospital Basel according to the Ethical approval obtained from the local Ethics Committee of Northwestern and Central Switzerland (EKNZ; Project-ID 2025-00588).

## Abbreviations

aSDH: Acute subdural hematoma
AUC: area under the receiver operating characteristic curve
BHD: Burr-Hole Drainage
CCI: Charlson Comorbidity Index
CI: Confidence interval
cSDH: Chronic subdural hematoma
CT: Computed tomography
DOAC: Direct oral anticoagulant
GCS: Glasgow Coma Scale
IQR: Interquartile range
MLS: Midline shift
MMAE: Middle meningeal artery embolization
MRI: Magnetic resonance imaging
mRS: Modified Rankin Scale
OR: Odds ratio
RCT: Randomized controlled trial

## Declarations

All patient data were handled in compliance with applicable data protection regulations.
