# Supplementary material for: Risk factors for the recurrence of chronic subdural hematomas in patients undergoing burr-hole drainage
Source: Neurosurg Rev. 2026 Nov 9;49(1):640. doi: 10.1007/s10143-026-04559-5 (PMC13745412; doi:10.1007/s10143-026-04559-5)
Supplement: Supplementary file 1 [file 10143_2026_4559_MOESM1_ESM.docx]

*Supplementary Table S1: Multivariable comparison of primary (imputed) analysis and complete-case sensitivity analysis.*

| **Variable** | **Primary Imputed Analysis (N = 765)** | **Complete-Case Sensitivity Analysis (N = 263)** |
| --- | --- | --- |
| **Intercept** | 0.007 (0.002–0.019); p < 0.001 | 0.054 (0.013–0.219); p < 0.001 |
| **Hypertension (No)** | 1.937 (1.167–3.213); p = 0.012 | 1.489 (0.685–3.237); p = 0.340 |
| **Charlson Comorbidity Index** | 1.003 (0.908–1.108); p = 0.955 | 0.927 (0.790–1.086); p = 0.373 |
| **Preoperative GCS (9-12)** | 1.249 (0.609–2.561); p = 0.557 | 1.540 (0.527–4.500); p = 0.452 |
| **Preoperative GCS (<8)** | 0.689 (0.175–2.715); p = 0.601 | 0.806 (0.110–5.891); p = 0.840 |
| **Preop Markwalder (Grade 2-3)** | 1.224 (0.699–2.145); p = 0.493 | 0.932 (0.381–2.282); p = 0.884 |
| **Preoperative Hematoma Volume** | 1.001 (0.999–1.003); p = 0.358 | 1.001 (0.998–1.004); p = 0.403 |
| **Preoperative Midline Shift** | 1.005 (0.989–1.021); p = 0.589 | 1.023 (0.981–1.067); p = 0.364 |
| **Hematoma Age (Acute on chronic)** | 1.136 (0.601–2.147); p = 0.703 | 1.617 (0.584–4.475); p = 0.378 |
| **Hematoma Age (Subacute)** | 2.097 (1.072–4.102); p = 0.036 | 2.401 (0.781–7.384); p = 0.146 |
| **Hematoma Age (Hygroma)** | 1.695 (0.454–6.324); p = 0.458 | 1.130 (0.150–8.522); p = 0.910 |
| **Hematoma Char (Laminar)** | 0.697 (0.298–1.632); p = 0.409 | 0.588 (0.159–2.178); p = 0.442 |
| **Hematoma Char (Separated)** | 0.974 (0.482–1.967); p = 0.942 | 0.967 (0.327–2.855); p = 0.954 |
| **Hematoma Char (Trabecular)** | 0.577 (0.278–1.199); p = 0.148 | 0.522 (0.163–1.673); p = 0.296 |
| **Postoperative Cavity Volume** | 1.012 (1.003–1.021); p = 0.016 | 1.007 (0.998–1.017); p = 0.136 |
| **Postoperative Midline Shift** | 1.179 (1.090–1.274); p < 0.001 | 1.032 (1.004–1.060); p = 0.018 |
| **Postoperative GCS (9-12)** | 0.579 (0.183–1.840); p = 0.363 | 0.852 (0.194–3.738); p = 0.840 |
| **Postoperative GCS (<8)** | 0.060 (0.002–1.692); p = 0.071 | 0.965 (0.026–35.257); p = 0.985 |
| **Postop Markwalder (Grade 2-3)** | 0.851 (0.384–1.888); p = 0.670 | 1.586 (0.500–5.030); p = 0.461 |
| **mRS at discharge (Group 3-5)** | 0.481 (0.184–1.258); p = 0.148 | 1.842 (0.391–8.672); p = 0.464 |
| **mRS Score at discharge** | 1.959 (1.304–2.944); p = 0.002 | 1.022 (0.539–1.937); p = 0.951 |
| **Apparent Discrimination (AUC)** | 0.809 | 0.746 (95% CI: 0.645–0.847) |
| **Hosmer–Lemeshow Test (p-value)** | p = 0.534 | p = 0.400 |
